# A single biochemical activity underlies the pleiotropy of the aging-related protein CLK-1

**DOI:** 10.1038/s41598-017-00754-z

**Published:** 2017-04-12

**Authors:** Ju-Ling Liu, Callista Yee, Ying Wang, Siegfried Hekimi

**Affiliations:** Department of Biology, McGill University, Montreal, H3A 1B1 Canada

## Abstract

The *Caenorhabditis elegans clk-1* gene and the orthologous mouse gene *Mclk1* encode a mitochondrial hydroxylase that is necessary for the biosynthesis of ubiquinone (UQ). Mutations in these genes produce broadly pleiotropic phenotypes in both species, including a lengthening of animal lifespan. A number of features of the *C*. *elegans clk-1* mutants, including a maternal effect, particularly extensive pleiotropy, as well as unexplained differences between alleles have suggested that CLK-1/MCLK1 might have additional functions besides that in UQ biosynthesis. In addition, a recent study suggested that a cryptic nuclear localization signal could lead to nuclear localization in cultured mammalian cell lines. Here, by using immunohistochemical techniques in worms and purification techniques in mammalian cells, we failed to detect any nuclear enrichment of the MCLK1 or CLK-1 proteins and any biological activity of a *C*. *elegans* CLK-1 protein devoid of a mitochondrial localization sequence. In addition, and most importantly, by pharmacologically restoring UQ biosynthesis in *clk-1* null mutants we show that loss of UQ biosynthesis is responsible for all phenotypes resulting from loss of CLK-1, including behavioral phenotypes, altered expression of mitochondrial quality control genes, and lifespan.

## Introduction

**Figure 1:**
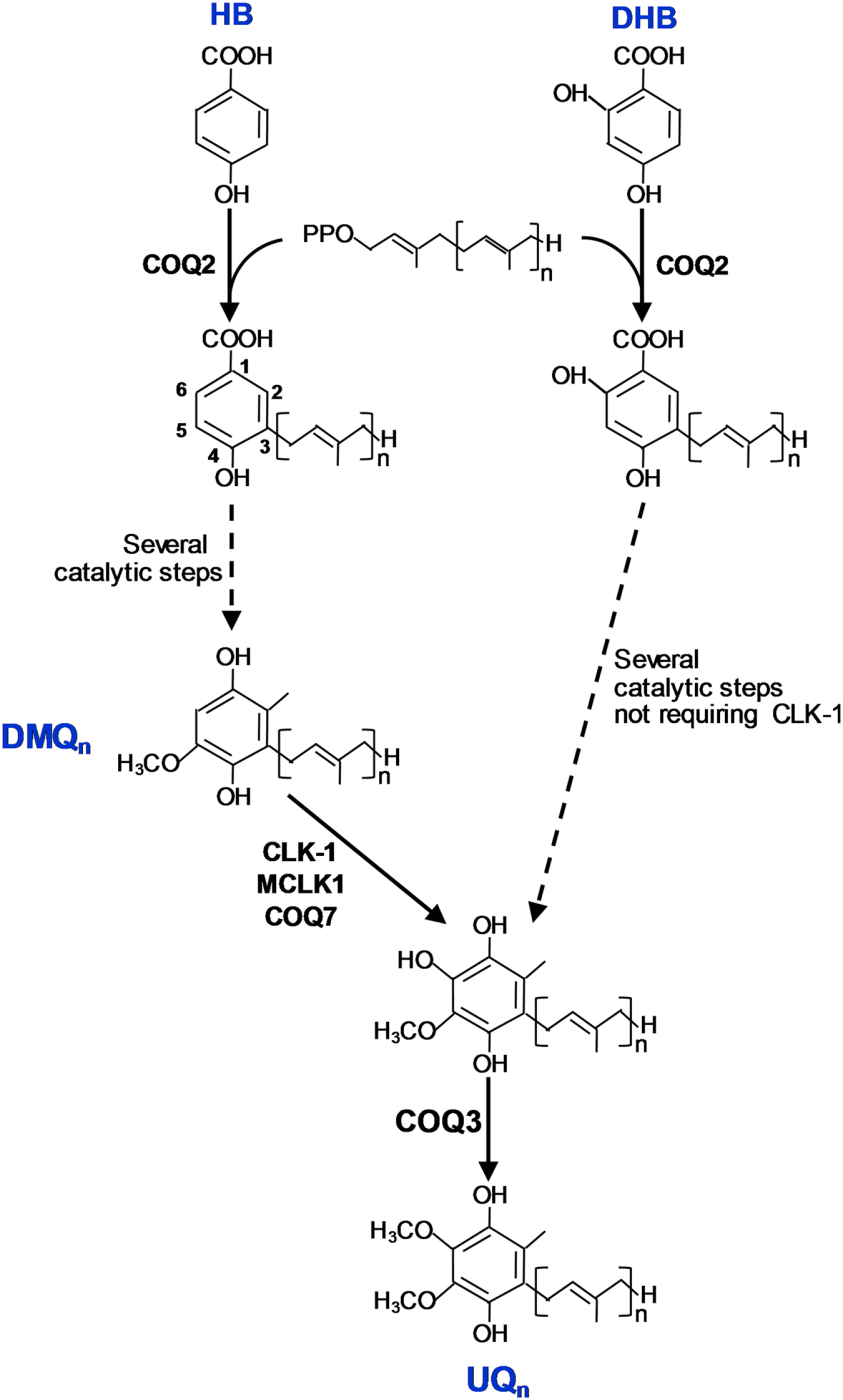
2,4-dihydroxybenzoate (DHB) is an unnatural bypass precursor of UQ biosynthesis. UQ is composed of a benzoquinone ring attached to a polyisoprenoid side chain. The length of the side chain varies between species. It is 9 isoprenoid subunits long in *C*. *elegans* (UQ_9_). All cells depend on endogenous biosynthesis for their supply of UQ. The enzyme encoded by *clk-1* and its orthologues (*Coq7* in yeast, *Mclk1* in mice, and *COQ7* in humans) catalyzes the penultimate step in UQ biosynthesis, the hydroxylation of DMQ at position 6. In eukaryotes, the natural biosynthetic precursor for the benzoquinone ring is 4-hydroxybenzoate (4-HB), but 2,4-dihydroxybenzoate (DHB), a hydroxylated analogue of 4-HB, is able to serve as an alternative, albeit unnatural, precursor of UQ synthesis that allows for the biosynthesis of UQ in the absence of CLK-1/COQ7/MCLK1 step^23, 24^.

The *C*. *elegans clk-1* mutant was among the very first long-lived mutants to be described and characterized molecularly^1, 2^. *clk-1* encodes a mitochondrial hydroxylase necessary for the biosynthesis of ubiquinone (UQ) (Fig. 1), an obligate electron transporter of the mitochondrial electron transport chain (ETC), and *clk-1* mutants have impaired mitochondrial function^3^. The longevity of *clk-1* mutants was the first observation connecting mitochondrial dysfunction to increased lifespan, a phenomenon that is now widely studied^4^. The mutant’s viability depends on obtaining UQ_8_ from the bacteria on which it feeds and mutants are consequently unable to grow on bacteria that are deficient in UQ biosynthesis^5–7^. The subscript in the abbreviation for UQ refers to the length of the isoprenoid side chain of UQ and is species-specific. *C*. *elegans* makes UQ_9_, *E*. *coli* UQ_8_ and mice predominantly UQ_9_ with some UQ_10 _^8^.

Bacterial UQ_8_ is unable to functionally replace endogenous UQ_9_ fully as *clk-1* mutants display a broadly pleiotropic phenotype. In addition to the increased lifespan, embryonic, post-embryonic, and germline development as well as rhythmic behaviours, such as the pumping and defecation cycles, are slowed down in *clk-1* mutants on average, and also profoundly deregulated^1, 9^. Embryonic development and defecation also show an inability to respond to changes in temperature^1, 10, 11^. In addition, and in contrast to the wild type, the defecation cycle of the mutants is sensitive to the level of cholesterol supplementation and to the presence of endogenous and exogenous bile acids^12^.

Homozygous *clk-1* mutants can be phenotypically rescued by the action of a wild type allele in their hermaphrodite parent^1^. Together with the complexity of the phenotype, these genetic observations have suggested that the action of maternal CLK-1 could induce an epigenetic state that alters gene expression^13^. However, subsequent work indicated that provisioning of the oocyte with maternally-derived CLK-1 and UQ mostly likely underlies the maternal effect^14^.

The two most studied alleles of *clk-1* are the null allele *qm30* and the phenotypically weaker missense allele *e2519*. All *clk-1* phenotypes are observed in both mutants but are less severe in the *e2519* mutant^1^. Despite their difference in severity, any possible differences in UQ biosynthesis are below the threshold of detection as endogenous UQ cannot be detected in either allele^15^. This could suggest that both alleles are null for UQ biosynthesis, but that *e2519* might have residual activity for some other, unknown, function.

Another observation suggesting an alternative function for CLK-1 comes from study of the anti-neurodegenerative divalent metal chelator clioquinol^16^. Clioquinol inhibits mammalian CLK-1 (MCLK1 in mice and COQ7 in humans) in cultured cells, presumably by preventing the binding of the two prosthetic iron atoms that are necessary for the catalytic function of CLK-1^17^. Treatment of *C*. *elegans* with clioquinol mimics several aspects of the *clk-1* phenotype, in particular the inability to grow on bacteria that are deficient in UQ biosynthesis, yet does not significantly prevent UQ synthesis, suggesting a possible function of CLK-1 in UQ transport^16^.

Most recently Monaghan *et al*. have suggested that CLK-1 could have a nuclear function that is independent of its role in ubiquinone biosynthesis. They found that mammalian COQ7 harbors a cryptic nuclear localization signal that, in transformed cell lines, results in a pool of nuclear, chromatin-associated COQ7 in addition to a mitochondrial pool^18^. The possibility of a nuclear role for CLK-1/MCLK1/COQ7 was supported by findings suggesting that the *C*. *elegans* CLK-1 protein devoid of its normal mitochondrial localization signal might partially rescue the extended mutant lifespan and the altered expression of genes involved in mitochondrial quality control^18^. This is a potentially interesting finding as a number of studies^19, 20^, albeit not all^21^, have suggested that long-lived *C*. *elegans* mitochondrial mutants require the function of genes involved in mitochondrial quality control, including the UPR^mt^ ^22^. However, it remains unclear what function would be served by the inhibition of mitochondrial quality control by nuclear wild-type CLK-1. The absence of nuclear COQ7 in transformed mammalian cells appears to affect cell growth, but the mechanisms of this effect have not been explored. Moreover, they did not test whether CLK-1 also localises to the nucleus in animal tissues or primary cells.

Here we directly address the question of whether CLK-1 has more than one function. We demonstrate that all tested aspects of the phenotype resulting from loss of *C*. *elegans* CLK-1, including rhythmic behaviors, increased lifespan and changes in gene expression, are due to loss of UQ biosynthesis. Furthermore, using biochemical and immunocytochemical methods we find no evidence for specific nuclear localization of CLK-1 or MCLK1, nor any activity associated with a CLK-1 protein devoid of mitochondrial localization signal. Thus, we conclude that CLK-1 has a single biological function, which is UQ biosynthesis. We discuss how, given the known properties and functions of UQ, altered UQ metabolism in *clk-1* mutants could account for their complex phenotype.

## Results

### Defective UQ biosynthesis in *clk-1* mutants is rescued by the unnatural precursor 2,4-dihydroxybenzoic acid (DHB)

The enzyme encoded by *clk-1* and its orthologues (*Coq7* in yeast, *Mclk1* in mice, and *COQ7* in humans) catalyzes the penultimate step in UQ biosynthesis, hydroxylating the benzoquinone ring of the biosynthetic intermediate demethoxyubiquinone (DMQ) at position 6 (Fig. 1). Consequently, *clk-1* mutants accumulate DMQ_9_ instead of UQ_9_, rely on UQ_8_ from their bacterial food source for viability, and cannot grow on *E*. *coli* strains that do not synthesize UQ, such as GD1^5, 7^. In eukaryotes, the natural biosynthetic precursor for the benzoquinone ring is 4-hydroxybenzoate (4-HB), but the 4-HB analogue 2,4-dihydroxybenzoate (DHB) (Fig. 1) is able to serve as alternative, albeit unnatural, precursor for UQ synthesis in yeast as well as in mammalian cells^23, 24^. For example, feeding *Mclk1* KO mice with DHB partially restores UQ biosynthesis and dramatically rescues their near-lethal phenotype^24^.

**Figure 2:**
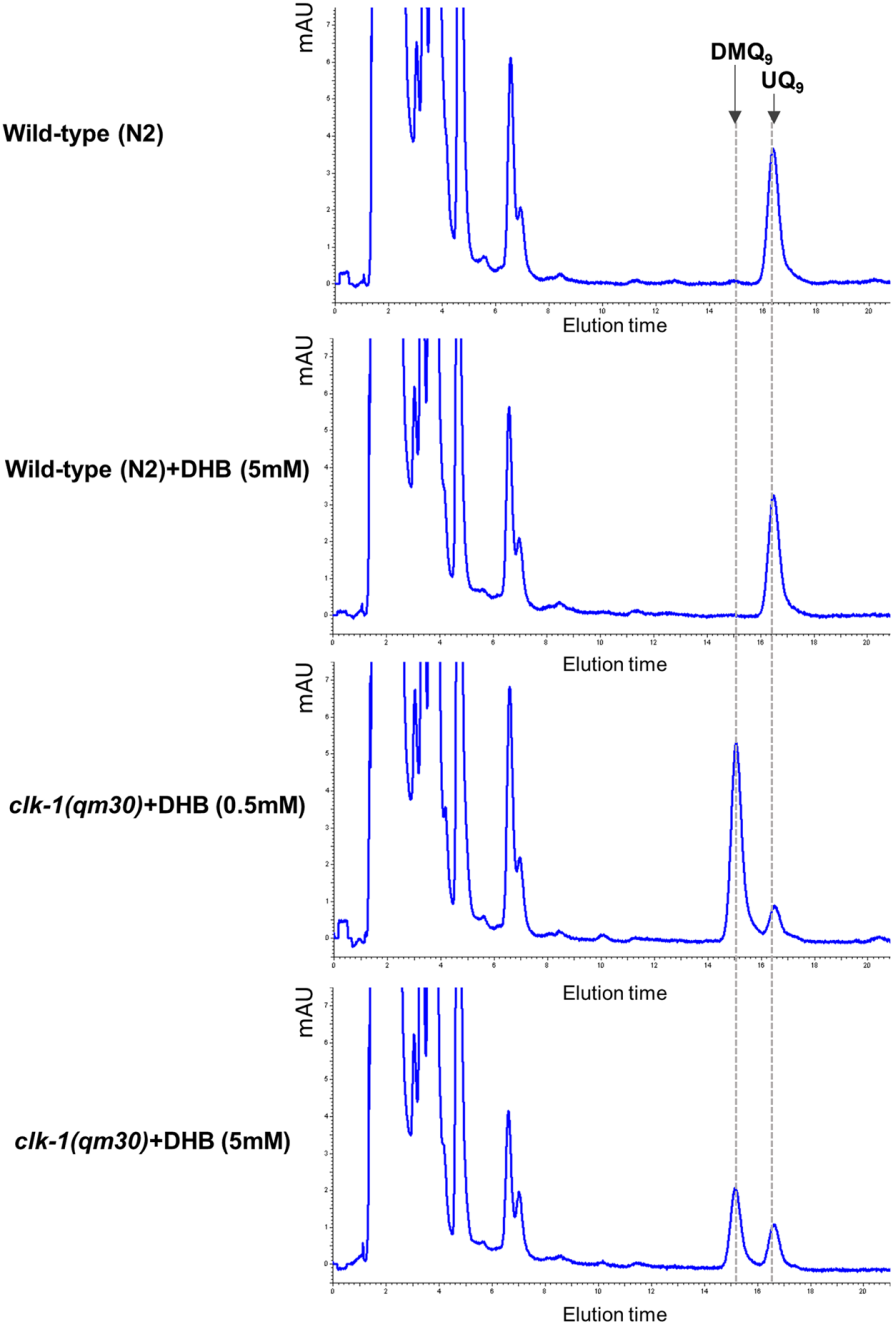
2,4-DHB supplementation restores UQ biosynthesis in *clk-1* mutants. HPLC-UV chromatograms of quinone extracts from worms with the indicated genotypes. Elution times for DMQ_9_ and UQ_9_ are indicated. At the first larval stage, the worms were transferred onto plates supplemented, or not, with DHB. They were fed the UQ-deficient bacterial strain GD1 and grown to the adult stage before being harvested for quinone extraction. Whole worm homogenates with 250 µg of protein were used, expect for the *clk-1*(*qm30*) mutants exposed to DHB at 0.5 mM in the plate where a homogenate with 440 µg of protein was used for HPLC analysis. Treatment with DHB restored UQ biosynthesis in *clk-1*(*qm30*) mutants in a dose-dependent manner, but showed no significant effect on the wild-type (N2).

We grew *C*. *elegans* wild type and *clk-1*(*qm30*) null mutants on UQ-deficient GD1 bacteria in the presence or absence of DHB. The mutant cannot grow at all on GD1 without DHB supplementation. But supplementation allowed worms to grow to adulthood. The rescue of growth by DHB was already complete with 0.5 mM, with both the wild type animals and the treated *clk-1* mutants already having grown to adulthood and produced L1 larval progeny on day 4 after hatching (Table S1). DHB produced two dose-dependent effects on quinone synthesis in these worms (Fig. 2): partial restoration of UQ levels and inhibition of DMQ levels, similar to what is observed in mammals^24^. The decrease of DMQ levels produced by DHB is believed to be due to competition with the native (4-HB-dependent) pathway^24^. As expected DHB is without effect on the wild type (Fig. 2).

### Full rescue of behavioral phenotypes by DHB

Having shown that DHB functions in worms as in mammals (Fig. 2), we reasoned that any phenotype that can be suppressed by providing DHB to the mutant worms must be the result of the defect in UQ biosynthesis. Previous observations had shown that a very small amount of UQ_9_ in the presence of a large amount of DMQ_9_ is sufficient for viability on GD1^15^. Indeed, virtually full rescue of all mutant phenotypes had previously been obtained by restoration of a minimal level of UQ biosynthesis by suppression of the missense allele *clk-1*(*e2519*)^15^. However, suppression by a suppressor tRNA cannot distinguish between suppression by restoration of a sufficient amount of UQ synthesis and suppression by restoration of the synthesis of a small amount of wild type CLK-1 protein (by insertion of the correct amino acid by the suppressor tRNA). Thus the experiments with suppressor tRNA could not exclude that some phenotypic features of *clk-1* mutants are due to the loss of another function of CLK-1 besides UQ biosynthesis.

**Figure 3:**
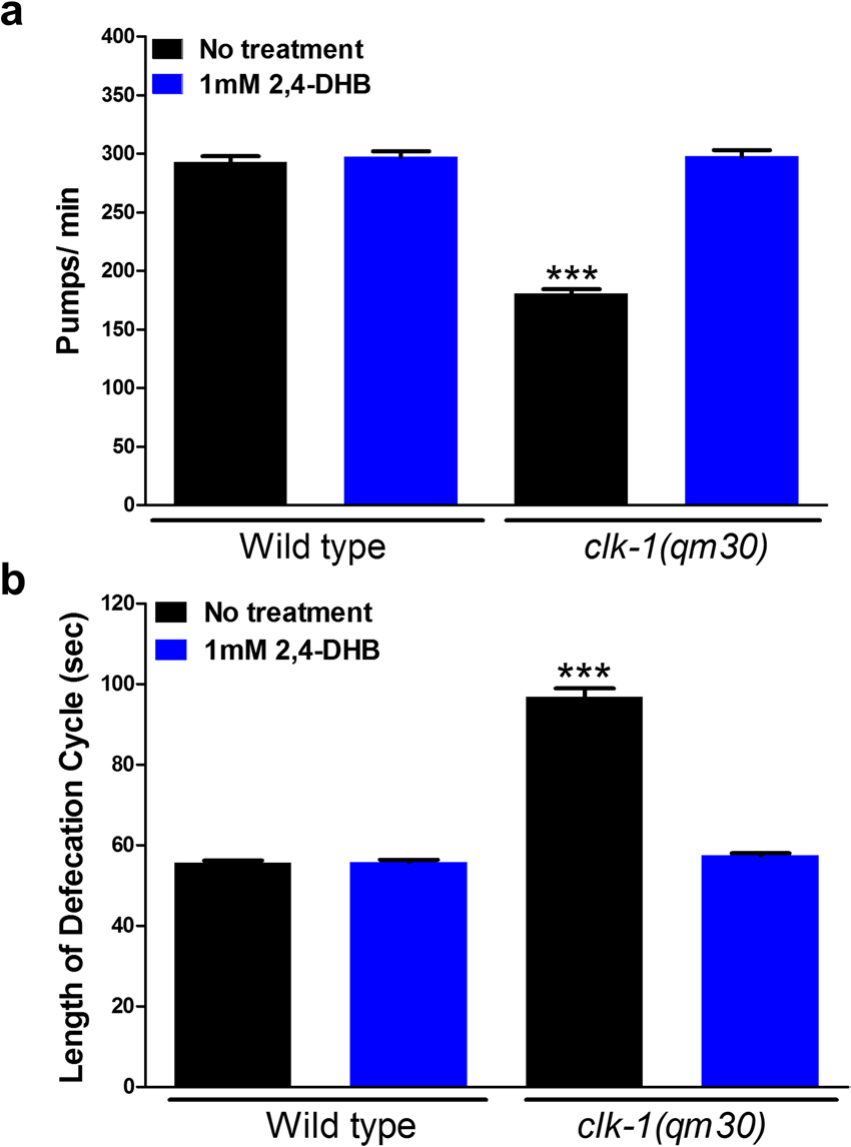
The behavioural defects of *clk-1*(*qm30*) mutants are fully rescued by 2,4-DHB. (**a**) *clk-1* mutants pump at a significantly slower rate than the wild type. 1 mM 2,4-DHB fully rescues the slow pumping rates of *clk-1*. At this concentration, 2,4-DHB had no effect on the wild type. The bars represent the mean number of pumps per minute. (**b**) *clk-1* mutants have a significantly lengthened defecation cycle length. 1 mM 2,4-DHB fully rescues the slow defecation phenotype of *clk-1*. 2,4-DHB had no effect on the wild type. The bars represent the mean defecation cycle of animals that have been scored for three consecutive defecation cycles each in the case of non-treated *clk-1*(*qm30*) mutants and for five consecutive defecation cycles for all the other conditions. The error bars represent S.E.M. (n ≥ 20 animals for each condition). The asterisks indicate that the data are significantly different from that of the wild-type. All differences were significant at p < 0.0001 by a t-test.

*C*. *elegans* have a highly regulated defecation phenotype that involves a series of muscular contractions followed by a waiting period before they are repeated. This behavior has been widely studied^10^, in particular detail in aging mutants^3, 21, 25, 26^, and an increased and irregular waiting period is a hallmark of *clk-1* mutants, even when grown on UQ-replete bacteria^1^. The regular pumping contractions of the worm’s feeding organ, the pharynx, are also affected by *clk-1* mutations, with fewer pumps per minute in the mutants^1^. We scored both behaviors in the presence of DHB, and found 1 mM could fully rescue the mutant phenotypes indicating that these phenotypes are due to the defect in UQ biosynthesis (Fig. 3).

### Full rescue of the *clk-1* mutant longevity by DHB

**Figure 4:**
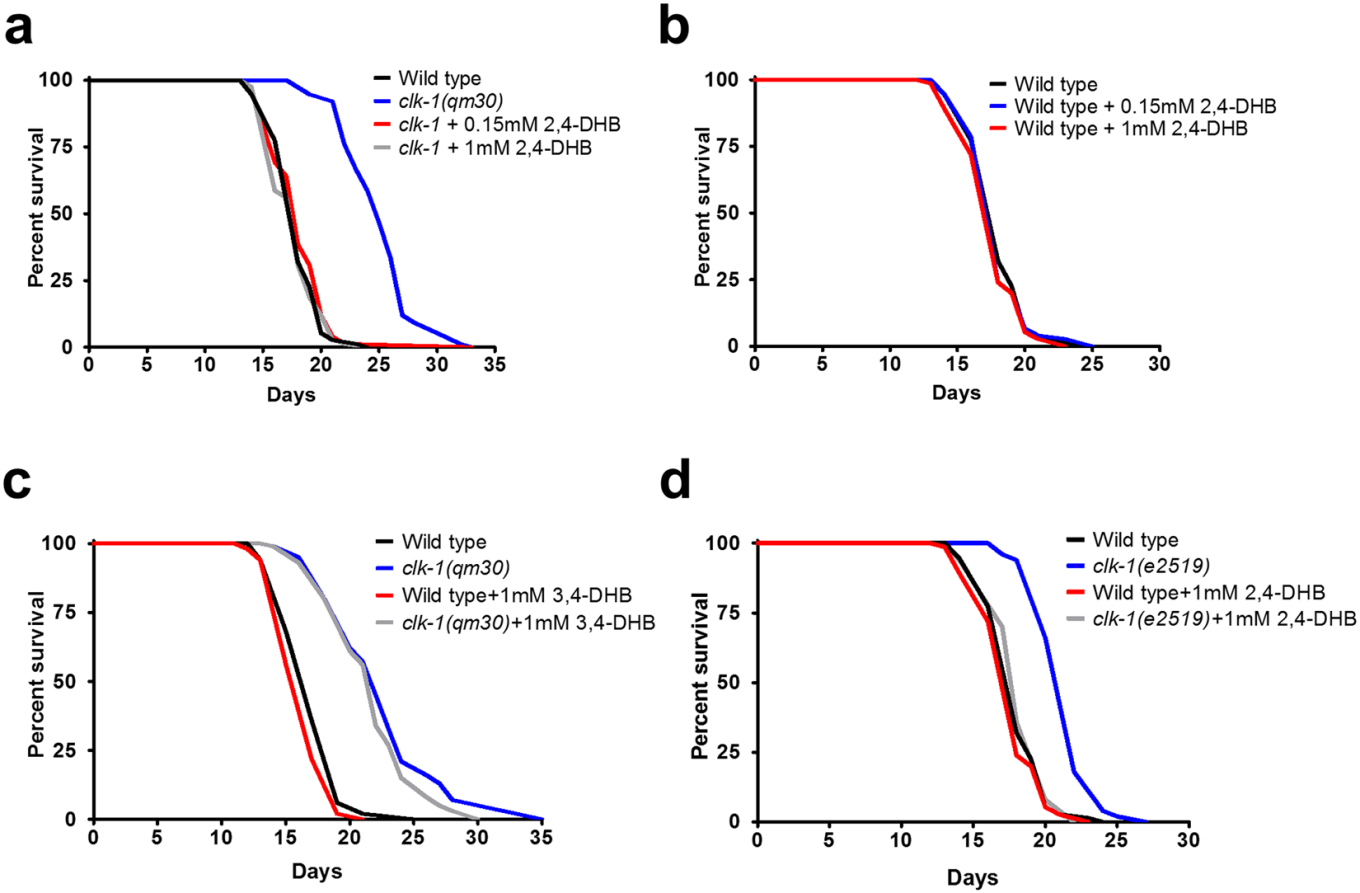
The longevity of *clk-1* mutants is rescued by 2,4-DHB. (**a**) Supplementation with 0.15 mM and 1 mM 2,4-DHB fully rescues the lengthened lifespan of *clk-1* mutants. (**b**) Supplementation with either 0.15 mM or 1 mM 2,4-DHB has no effect on the wild type lifespan. (**c**) Treatment with 1 mM 3,4-DHB does not affect the lifespan of either *clk-1* mutants or the wild-type. (**d**) Treatment with 1 mM 2,4-DHB rescues the lengthened lifespan of *clk-1*(*e2519*) mutants and has no effect on wild type lifespan. Sample sizes, numerical values and statistical analyses for all lifespan experiments are presented in Table S2.

The long life of *clk-1* mutants is one of their most interesting phenotypes. We found that restoring UQ biosynthesis with DHB fully suppresses the extended longevity of the canonical null allele *clk-1*(*qm30*) even with very low levels of supplementation (0.15 mM and 1 mM) (Fig. 4a). No effect on the lifespan of the wild type was observed (Fig. 4a). We also carried out the following additional controls. First, in contrast to DHB, which is 2,4-dihyroxybenzoate, we did not observe any effect on lifespan of 3,4-dihydroxybenzoate (3,4-DHB) (Fig. 4c), an almost identical compound that however cannot be used to by-pass the absence of CLK-1 (Fig. 1)^8^, indicating that all the effects are specific to restored UQ synthesis. Second, DHB also fully suppressed the extended lifespan of *clk-1*(*e2519*), a widely-studied partial loss of function allele producing a stable full length protein^27^ (Fig. 4d). These results indicate that the longevity phenotype of *clk-1* mutants is also entirely due to the defect in UQ biosynthesis.

### No rescue of the *clk-1* mutants’ developmental, behavioral and aging phenotypes by a CLK-1 protein devoid of mitochondrial targeting sequence

**Figure 5:**
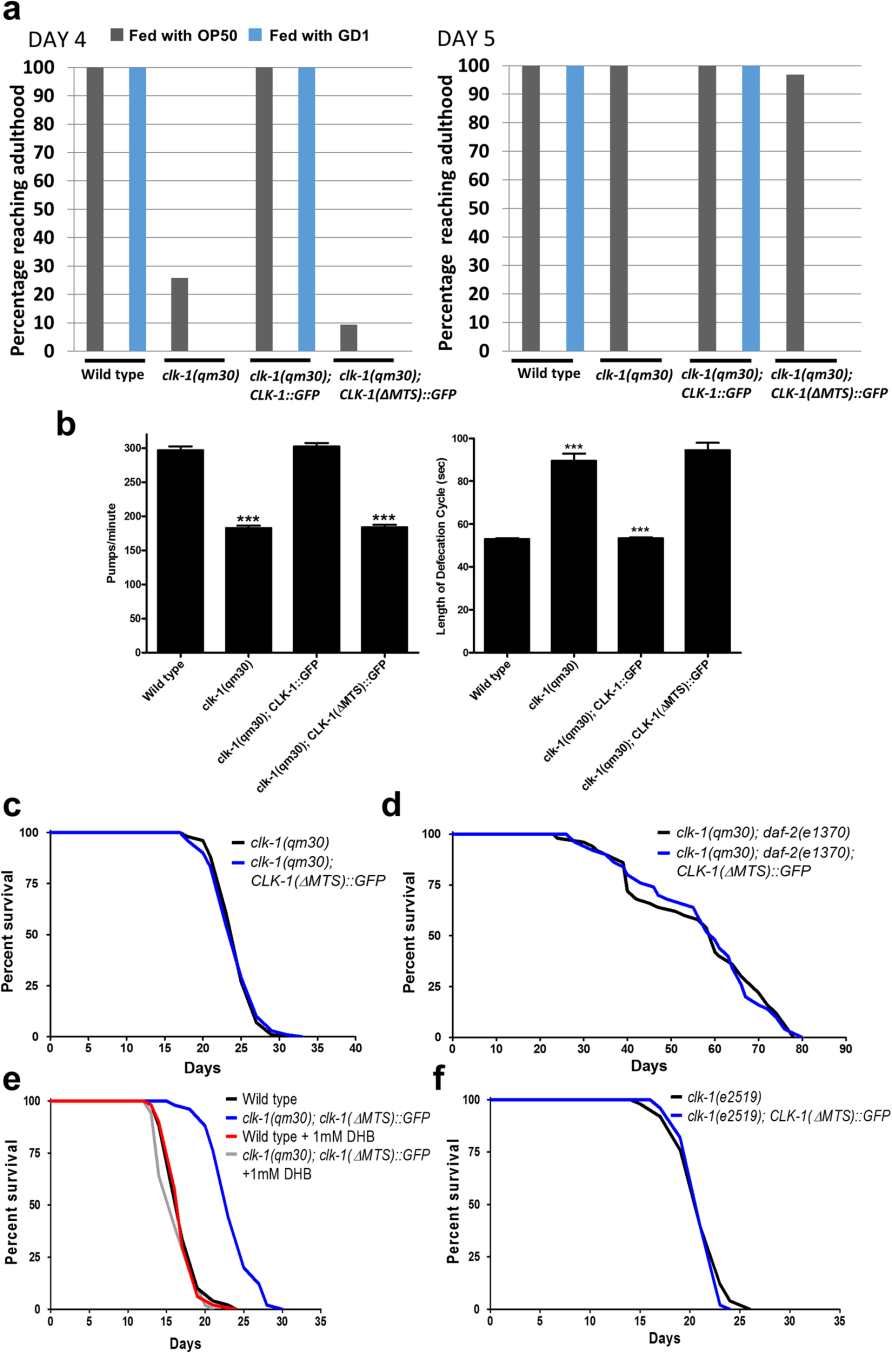
*CLK-1*(*ΔMTS*)::*GFP* cannot rescue the phenotypes of *clk-1* mutants (**a**) *clk-1*(*qm30*) mutants grow slowly when fed OP50 and arrest when fed UQ_8_-deficient bacteria GD1. Both phenotypes are rescued by a single copy insertion of full length CLK-1::GFP. Neither phenotype is rescued by a single copy insertion of *CLK-1*(*ΔMTS*)::*GFP*, which lacks the mitochondrial targeting sequence of *clk-1*. (n ≥ 30 animals for each genotype). (**b**) The slow pumping rate and slow defecation of *clk-1*(*qm30*) mutants can be fully rescued by the expression of full length CLK-1::GFP but not by the expression of CLK-1(ΔMTS)::GFP. Bars represent the mean number of pumps per minute or the mean defecation cycle of animals that have been scored for three consecutive defecation cycles each in the case of *clk-1*(*qm30*) mutants and for five consecutive defecation cycles for all the other genotypes. The error bars represent S.E.M. (n ≥ 20 animals for each genotype). The asterisks indicate that the data are significantly different from that of the wild-type. All differences were significant at p < 0.0001 by a t-test. (**c**), (**d**) and (**f**) The expression of CLK-1(ΔMTS)::GFP had no effect on the lifespans of *clk-1*(*qm30*), *clk-1*(*qm30*); *daf-2*(*e1370*) or *clk-1*(*e2519*) mutants. (**e**) Treatment with 1 mM 2,4-DHB rescues the lengthened lifespan of *clk-1*(*qm30*); *CLK-1*(*ΔMTS*)::*GFP* and has no effect on the lifespan of the wild type. Numerical values and statistical analyses for all lifespan experiments are presented in Table S2.

To test the possibility that CLK-1 acts in the nucleus, Monaghan *et al*. used the MosSCI technique^28^ to obtain strains that expressed a single copy of either of two forms of CLK-1 from a genomic site distinct from the *clk-1* locus, but under the control of *clk-1* regulatory sequences^18^. The two forms are the wild-type CLK-1 sequence fused to GFP (CLK-1::GFP), and a CLK-1 sequence without its normal mitochondrial targeting sequence, also fused to GFP (CLK-1(ΔMTS)::GFP). The authors kindly sent us two strains with these transgenic loci in an otherwise wild-type background (strains OL0092 ukSi1[*pclk-1*::*clk-1*::*gfp*] and OL0119 ukSi2[*pclk-1*::*clk-1ΔMTS*::*gfp*]). We transferred these loci into the *clk-1*(*qm30*) genetic background and scored *clk-1* phenotypes. We observed that *clk-1*(*qm30*) only makes DMQ and no UQ as expected, and that this can be rescued by transgenic CLK-1::GFP, as has also been found earlier with an extrachromosomal multi-copy transgene^3^, but not by transgenic CLK-1(ΔMTS)::GFP (Fig. S1). We also found that CLK-1::GFP, but not CLK-1(ΔMTS)::GFP, allowed for growth on the UQ-deficient GD1 *E*. *coli* strain (Fig. 5a). Similarly, we found that expression of CLK-1::GFP fully rescued the slow defecation and slow pumping phenotypes, but that expression of CLK-1(ΔMTS)::GFP had no effect at all (Fig. 5b).

Most crucially we tested whether, like Monaghan *et al*., we could observe a difference in lifespan between *clk-1* and the strain expressing CLK-1(ΔMTS)::GFP in the *clk-1*(*qm30*) background. However, we did not observe any rescue of the increased lifespan (Fig. 5c). To test this question further we used the *clk-1*(*qm30*);*daf-2*(*e1370*) genotype. These two mutations act synergistically and the double mutants have a much increased lifespan compared to *daf-2* or *clk-1* alone^29^. Here we could recapitulate this effect with the double mutants having an average lifespan of 56 days and a maximum lifespan of 78 days (Fig. 5d), much longer than the published range for *daf-2* (30 to 49 days^30, 31^). A partial rescue of the aging-relevant activity of CLK-1 in this background could potentially reveal greater lifespan effects than in the *clk-1* background alone. We constructed a triple mutant strain that also contained the locus expressing CLK-1(ΔMTS)::GFP and scored lifespan in comparison to the double mutants (Fig. 5d). Again, we did not find any evidence of a rescue by the transgene expressing CLK-1(ΔMTS)::GFP.

We also carried out the following controls. We determined that 1 mM DHB was still fully capable of rescuing the extended longevity of *clk-1* in the *clk-1*(*qm30*); CLK-1(ΔMTS)::GFP strain (Fig. 5e), indicating that the longevity of this strain, which is not different from that of *clk-1*(*qm30*) (Fig. 5c and e) was still, and only, dependent on the status of UQ biosynthesis. We also determined that the longevity of *clk-1*(*e2519*) was not affected by the presence of CLK-1(ΔMTS)::GFP (Fig. 5f), indicating that the longevity of this mutant, which produces a stable full-length protein, again depends only on the status of UQ biosynthesis (Fig. 4d). All numerical data corresponding to the graphical representation of the figures so far are given in Table S2.

From all the observations above, we conclude that the CLK-1(ΔMTS)::GFP protein has no activity on the lifespan of *clk-1*(*qm30*) mutants and other *clk-1* alleles. Furthermore, given the mechanism of action of DHB (restoration of UQ synthesis), and its ability to fully rescue the longevity of *clk-1* mutants, we conclude that the increased lifespan of *clk-1* mutants is entirely due to their lack of endogenous UQ synthesis. This is in contrast to Monaghan *et al*., who observed a partial rescue of the longevity phenotype by CLK-1(ΔMTS)::GFP.

### Changes of gene expression resulting from loss of CLK-1 are rescued by DHB

**Figure 6:**
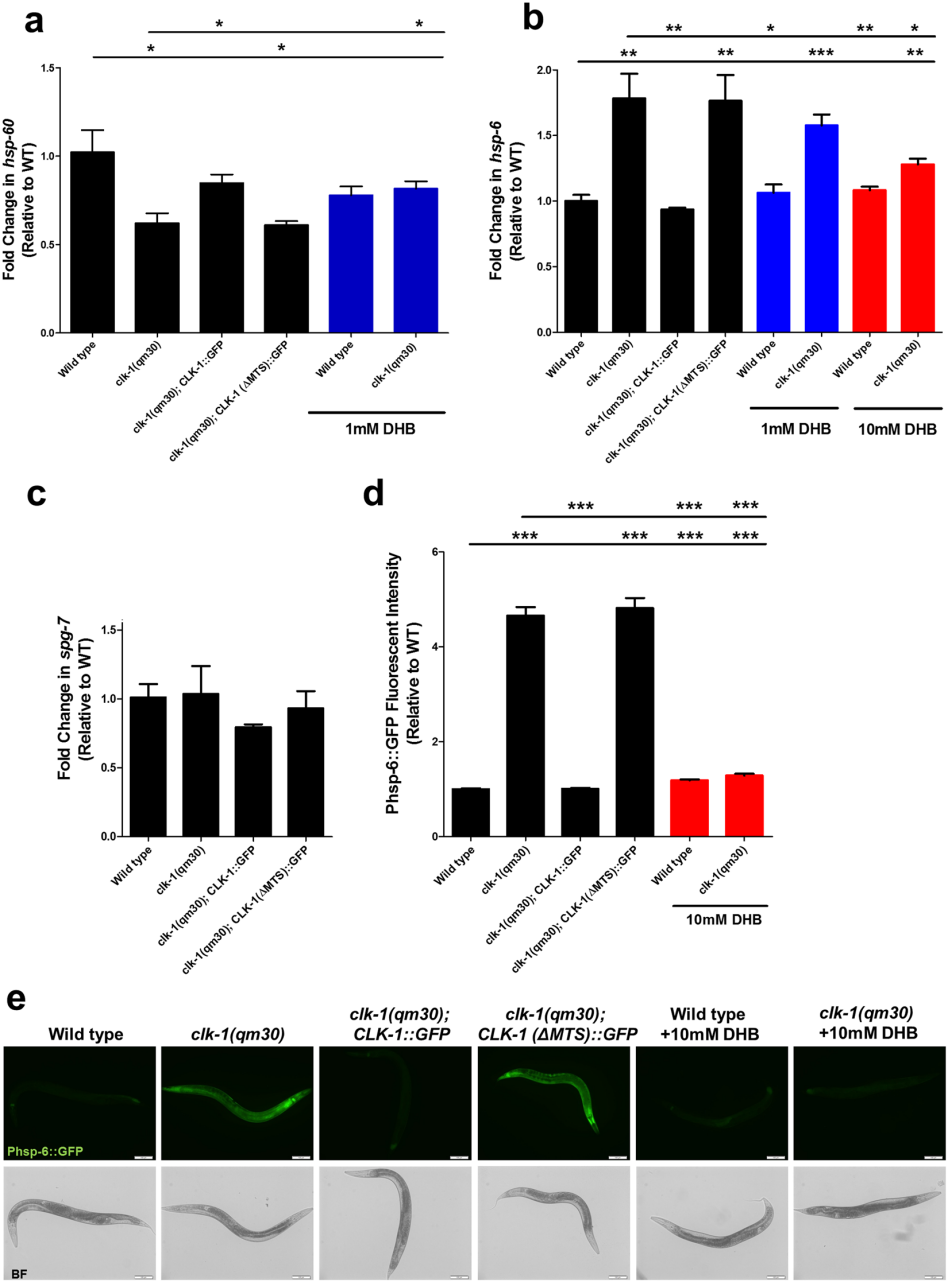
Changes in gene expression in *clk-1* mutants can be rescued by the expression of CLK-1::GFP or by 2,4-DHB treatment. (**a**,**b**) The mRNA level of UPR^mt^ genes was examined in day 1 adult worms by quantitative real-time PCR. Results were normalized to mRNA levels in wild type. *clk-1*(*qm30*) worms showed altered expressions of *hsp-60* and *hsp-6*. The expression of CLK-1::GFP, but not CLK-1(ΔMTS)::GFP, rescues the transcript levels of *hsp-60* and *hsp-6* in *clk-1* mutants. The same rescuing effect can be achieved by treating *clk-1* mutants with 2,4-DHB. (**c**) The *spg-7* mRNA levels were the same for the all genotypes that we examined. (**d**,**e**) Quantification of GFP intensity from *Phsp-6*::*GFP*. Values are expressed as fold change in GFP fluorescence intensities relative to the GFP fluorescence intensity of the *Phsp-6*::*GFP* reporter in the wild type background. The treatment of 2,4-DHB decreased the reporter expression in *clk-1* mutants, while it had only a small effect on the control strains. Error bars indicate S.E.M. (n ≥ 30 animals). *p < 0.05, **p < 0.01, ***p < 0.001. Scale bar: 100 µm.

Since Monaghan *et al*. suggested that the mitochondrial unfolded protein response (mtUPR) is activated by the absence of nuclear CLK-1, we quantified the expression of three genes (*spg-7*, *hsp-60* and *hsp-6*) by qPCR in the wild type, in *clk-1* mutants, and in *clk-1* mutants also expressing CLK-1::GFP or CLK-1(ΔMTS)::GFP (Fig. 6). The primers and control genes we used are shown in Table S3. We did not observe any effect of the *clk-1* mutation on the expression of *spg-7* (Fig. 6c). We observed that the expression of *hsp-60* was lowered by the mutation, and consistent with all our other observations, this lowering was rescued by expression of CLK-1::GFP but not by CLK-1(ΔMTS)::GFP (Fig. 6a). Furthermore, we found that treatment with 1 mM DHB of the wild type and *clk-1* mutants abolished the difference of *hsp-60* expression, suggesting that the difference observed without treatment results from the defective UQ metabolism in the mutants (Fig. 6a). We also observed that *hsp-6* expression was increased in the mutant (Fig. 6b), and like *hsp-60*, the expression level could be rescued by expression of CLK-1::GFP but not by CLK-1(ΔMTS)::GFP. For this gene we also monitored the expression of GFP reporter driven by the *hsp-6* promoter (*Phsp-6*::*GFP*) and expressed from a transgene^32^ and obtained the same result (Fig. 6e and d): that is, increased expression in *clk-1* mutants was abolished by CLK-1::GFP and not by CLK-1(ΔMTS)::GFP. Interestingly, we found that treatment with 1 mM DHB was insufficient to normalize the expression of *hsp-6* in *clk-1* mutants, instead 10 mM was necessary (Fig. 6b,d and e). Possibly altered *hsp-6* expression is particularly sensitive to UQ levels or is elevated in a deep tissue that is not easily reached by DHB. In any case, this greater sensitivity is unrelated to lifespan regulation, as 1 mM DHB was sufficient for full suppression of the increased longevity.

These results differ strikingly from those of Monaghan *et al*., who observed that *hsp-6*, *hsp-60*, and *spg-7* all had increased expression in *clk-1* mutants but not in *clk-1* mutants also expressing CLK-1(ΔMTS)::GFP^18^. They concluded that a potential nuclear activity of the full-length protein (in the wild type) and of the truncated protein is capable of regulating gene expression directly. This was a surprising finding as CLK-1(ΔMTS)::GFP, in contrast to the human homologue, has no nuclear localisation signal. Our findings about gene expression are consistent with our findings that all organismal phenotypes we have scored, including lifespan, are fully rescued by treatment with 1 mM DHB.

### Establishing the cellular distribution of a CLK-1 protein with or without a mitochondrial targeting signal

Previous studies by our group have consistently detected *C*. *elegans* CLK-1::GFP protein to be localized to the mitochondria^3^. However, Monaghan *et al*. report GFP expression in both mitochondria and nuclei in their single-copy *clk-1*::*gfp* transgenic strains^18^. Furthermore, they describe that deletion of the mitochondrial targeting sequence in CLK-1(ΔMTS)::GFP resulted in a profound increase in the number of GFP-positive nuclei. However, no quantification was shown.

Using fluorescent microscopy, we analyzed worms expressing CLK-1(∆MTS)::GFP and found it to be virtually undetectable in embryos, larval stages or adults. This was in contrast to CLK-1::GFP which was at least detectable in developing and adult animals (not shown). Since these two transgenes were driven by the same *clk-1* promoter, we wondered if this discrepancy between Monaghan *et al*.’s observations and our results was due to sequence changes at these loci in our strains. We re-sequenced both loci and found no sequence difference or error. Since the mitochondrial targeting sequence is presumably required for normal CLK-1 localization, it is expected that deletion of this sequence could result in diffuse expression and/or cause localization to other cellular compartments. Note that there is no nuclear localization sequence in CLK-1(ΔMTS)::GFP^18^.

To test these questions further, we performed immunohistochemistry to help with the visualization of CLK-1::GFP and CLK-1(∆MTS)::GFP. We stained worms at all developmental stages using antibodies against GFP and the mitochondrial marker HSP-60^33^. Stained slides were mounted with DAPI as a marker for nuclei. Whole embryo and worm sections were imaged and the co-localization of fluorescent signal (GFP vs. HSP-60 and GFP vs. DAPI) was evaluated by calculating a Pearson’s correlation coefficient (PCC). To ensure that the GFP fluorescence we observed was not an artifact of auto-fluorescence, we performed staining controls lacking either the primary or secondary antibodies. We observed negligible background in both FITC and TxRED channels (Fig. S2).

In both strains (expressing CLK-1::GFP or CLK-1(ΔMTS)::GFP), we observed a positive correlation of GFP with HSP-60 (Fig. S3A and Fig. S4A) and a negative correlation with DAPI (Fig. S3B and Fig. S4B). Our staining results do not exclude the possibility that some CLK-1 or CLK-1(∆MTS) is localized to the nucleus and performs some function. But, as the proteins are clearly not enriched in the nucleus, this would be true for virtually any protein expressed at low levels or protein that has lost its normal localization signal. Still, even the CLK-1(∆MTS)::GFP protein shows a positive correlation with HSP-60 and not with DAPI.

### Establishing whether mouse MCLK1 is enriched in nuclear fractions

**Figure 7:**
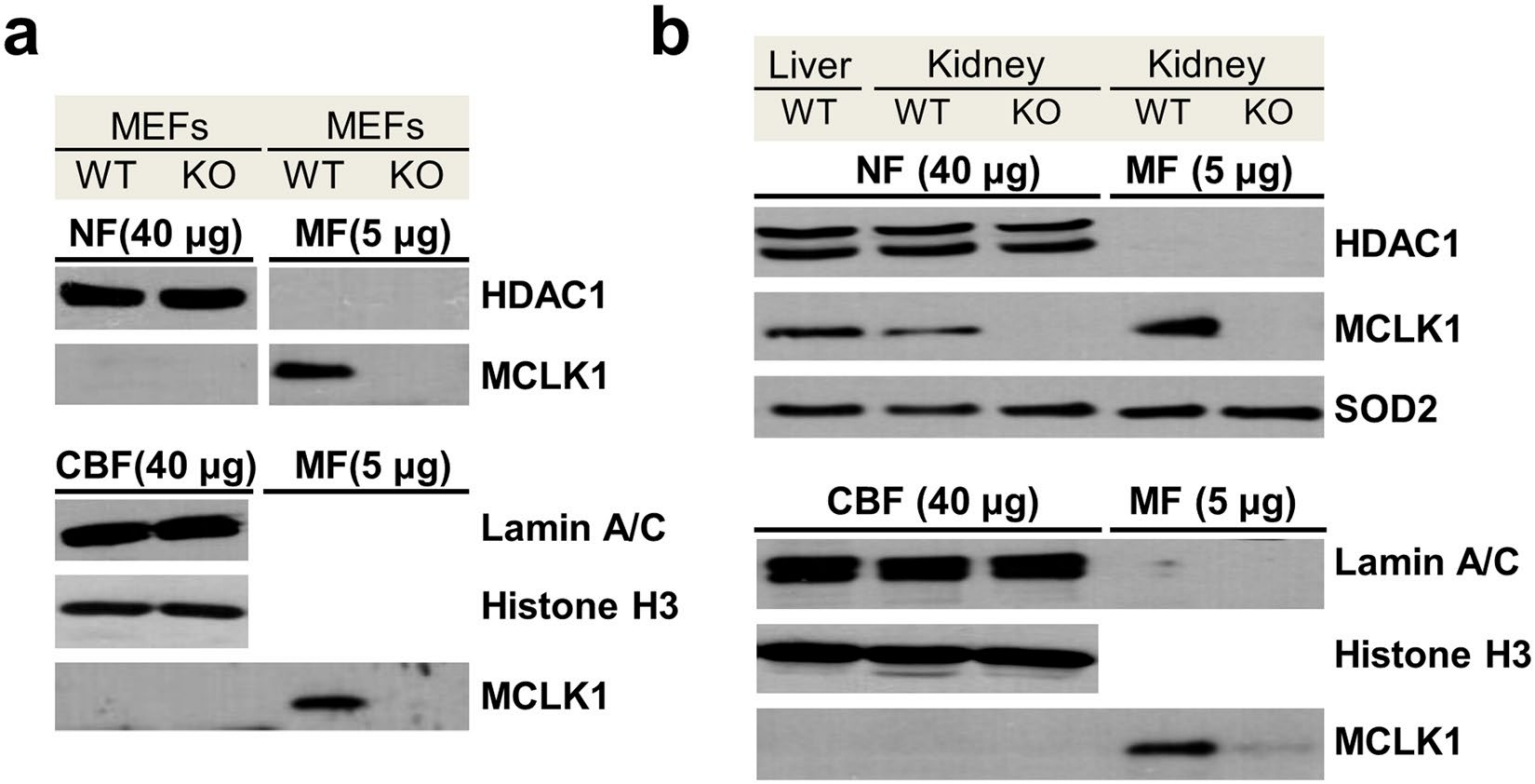
Detection of MCLK1 in protein fractions. Western blot analysis of nuclear protein extracts prepared from mouse embryonic fibroblasts (MEFs) (**a**) and Western blot analysis of nuclear protein fractions from mouse tissues (**b**). *Mclk1* KO cells were generated as previously published^34^ and the KO tissue samples were obtained from ago*Mclk1* KO mice described in ref. 47. They were used to further confirm band identity. The wild-type controls are *Mclk1* floxed MEFs or tissues without Cre expression. In both panels, 40 µg proteins per lane were loaded except for the membrane fraction (MF), where 5 µg were used. HDAC1 was used as a loading control of nuclear soluble fractions (NF). For insoluble (chromatin-bound) nuclear fractions (CBF), lamin A/C and Histone H3 served as markers. The mitochondrial matrix protein SOD2 was used as an additional control to validate the purity of the nuclear protein fractions. (**a**) In MEFs, MCLK1 could only be detected in membrane fractions (MF). (**b**) In mouse tissues, MCKL1 could be detected in both nuclear soluble fractions (NF) and membrane fractions (MF). However, the mitochondrial matrix protein SOD2 could also be detected in both fractions suggesting that the nuclear soluble fraction was contaminated by mitochondria. MCLK1 could not be detected in the chromatin-bound nuclear fraction (CBF). No evidence therefore points to the presence of MCLK1 in the nucleus or an association with chromatin. Uncropped western blot scans with size indications are shown in Supplementary Data.

To follow up on this, we determined whether we could detect mouse CLK-1 (MCLK1) in the nucleus. We obtained nuclear protein extracts from wild type and *Mclk1* KO mouse embryonic fibroblasts (MEFs)^34^, and sought to detect the presence of MCLK1 by immunoblotting. As shown in Fig. 7a, we detected MCLK1 in the membrane fraction (MF) of wild type cells but not *Mclk1* KO cells as expected. However, no MCLK1 was seen in the nuclear fraction (NF): neither in the soluble fraction (containing HDAC1) nor in the insoluble chromatin-bound fraction (CBF; containing Lamin A/C and histone H3). We also performed the same subcellular fractionation analysis on mouse tissues (Fig. 7b). MCLK1 was detected in the soluble nuclear fraction (containing HDAC1). However, this result is inconclusive, as the soluble mitochondrial protein SOD2 was also easily detected, indicating incomplete purity and unavoidable mitochondrial protein contamination in the soluble nuclear protein extracts. However, in the chromatin-bound insoluble nuclear fractions (CBF) prepared from the liver and kidneys of wild type mice we could not detect any MCLK1. This is in contrast to Monaghan *et al*. who detected tagged COQ7 in the mitochondria and in the nuclear chromatin fraction in transformed HEK293 cells.

## Discussion

The *C*. *elegans clk-1* mutant model of longevity was the first genetic link between altered mitochondrial function and longevity^2, 3^. It is frequently used to distinguish mitochondria-dependent longevity from other mechanisms. It is also a model to study UQ biology and how perturbations in reactive oxygen species (ROS) metabolism affect aging and various physiological features^8^, questions that are also studied in mice with mutants of the *clk-1* orthologue *Mclk1* ^35, 36^.

*clk-1* mutants, as well as other mitochondrial mutants, are also being used to explore the importance of the mitochondrial unfolded protein response (mtUPR) in the longevity mechanisms linked to mitochondrial dysfunction^4, 20, 37^. One debate is whether activation of mtUPR is necessary for mitochondrial dysfunction-dependent longevity and/or whether it is also sufficient^4, 38^. The interest of the study by Monaghan *et al*. can be placed in this context. Indeed, their observations suggested that a CLK-1 protein devoid of a mitochondrial targeting signal (CLK-1(ΔMTS)::GFP) can act in the nucleus to affect, in particular to decrease, the expression of genes involved in the mtUPR. Furthermore, this activity might be relevant to the aging process in *clk-1* mutants, as Monaghan *et al*. found that the presence of the *clk-1*(*ΔMTS*)::*gfp* transgene led to a lesser longevity increase of *clk-1* mutants.

However, we have found that treatment with the alternative UQ precursor (DHB), whose use by-passes the need for the hydroxylation normally performed by CLK-1, is sufficient to rescue all aspects of the phenotype of *clk-1* mutants that we have studied, including changes in gene expression. Furthermore, an almost identical molecule, 3,4-DHB, which can serve as an alternative unnatural precursor for yeast cells that lack the function of the UQ-biosynthetic Coq6p enzyme, but not for cells that lack the CLK-1 orthologue Coq7p^39–41^, has no effect on lifespan, nor any other phenotypic effects (not shown). This means that all phenotypes of *clk-1* mutants are linked to UQ biosynthesis, which is restored by 2,4-DHB (DHB). Thus if CLK-1 had a function in the nucleus this function would have to be the synthesis of UQ in that compartment. This would mean that all the other enzymes involved in modifying the aromatic ring of 4-HB would also have to localize to the nucleus, while to date they have only be detected in mitochondria^36^. Our findings of complete phenotypic rescue using the restoration of UQ biosynthesis with DHB in *clk-1* mutants thus imply that the pleiotropic phenotype of *clk-1* mutants is associated with the variety of functions of UQ, which is found in all biological membrane and is a co-factor for a number of enzymes involved in signaling events at the plasma membrane as well as in many aspects of mitochondrial function^36, 42^. In addition, transfer of electrons from and to UQ, including during signaling events at the plasma membrane, results in the generation of ROS, which are themselves pleiotropic signaling molecules^43^. In fact, the effect of altered ROS signaling on a variety of phenotype observed in *clk-1* mutants has been documented in several studies^9, 12, 44^.

We believe our analysis of the rescue of *clk-1* phenotypes by DHB is logically sufficient to demonstrate that the pleiotropy of the *clk-1* phenotype is entirely due to a defect in UQ biosynthesis. How to explain then the results by Monaghan *et al*.? We have explored this question by further characterizing the phenotypes associated with the two single copy transgenic strains constructed by these authors: one transgene producing a CLK-1::GFP protein, and the other a similar protein devoid of mitochondrial targeting sequence (CLK-1(ΔMTS)::GFP). We employed mostly different and a greater variety of tests of activity than those of Monaghan *et al*. However, we could detect no phenotypic effect produced by CLK-1(ΔMTS)::GFP in any of our tests, even those that were similar to those used by Monaghan *et al*., such as lifespan and gene expression. The strains with the single copy transgenes we received from the authors were in the wild type background. We have verified their sequences, which were identical to those described in Monaghan *et al*. We introduced them into the *clk-1*(*qm30*) background by mating. Thus, one possibility to explain the difference between our results and those of Monaghan is that the *clk-1* background in which they scored the effect of their transgenes contained additional genetic changes that produce *clk-1*-like phenotype that can be affected by the transgenes. In addition, it is always possible that subtle differences in animal husbandry or experimental protocols can yield different results. However, our findings with both DHB and the transgenes are fully consistent with the absence of any UQ-independent activity of the CLK-1 protein.

In addition, we have used different methodologies than those of Monaghan *et al*. to study the localization of full length CLK-1::GFP and CLK-1(ΔMTS)::GFP. In *C*. *elegans*, to overcome the very low level of expression, we have used antibody staining against GFP and analyzed the co-localization of this staining with antibody staining against a mitochondrial marker (HSP-60) and dye-staining for DNA (DAPI). The GFP staining is weak and diffuse for both transgenes. However, we found a positive correlation of the staining with mitochondrial staining and a negative correlation with nuclear staining for both transgenes. Although our findings cannot demonstrate that there is no amount of CLK-1::GFP or CLK-1(ΔMTS)::GFP in the nucleus, the absence of any nuclear enrichment still represents an absence of evidence that either of these proteins plays a biological role in this compartment.

Similarly, Monaghan *et al*., present data which they interpret as suggesting that COQ7 is partly nuclear and associated with chromatin in transformed human cell lines. To study this further, we have used an antiserum against MCLK1 to attempt to detect MCLK1 in the nuclear compartment in primary mouse cells (MEFs) and mouse tissues. No MCLK1 was found in the nuclear compartment of the primary cultured cells, and no MCLK1 was found associated with the insoluble (chromatin-bound) fraction in mouse tissue. Overall, our findings with primary mouse cells and mouse tissues suggest that the chromatin association observed by Monaghan *et al*. represents a transformed cell phenomenon.

## Methods

### General methods and strains

All animals were grown at 20 °C and cultured on regular NGM plates. The Bristol strain N2 was used as the wild type. The mutations used in this study were as follows: *clk-1*(*qm30*) III, *clk-1*(*e2519*) III and *daf-2*(*e1370*) III. The transgenic strains generated for the study were: MQ1964 ukSi1 [*pclk-1*::*clk-1*::*gfp*, *cb-unc-119*(+)]; *clk-1*(*qm30*), MQ1966 ukSi2 [*pclk-1*::*clk-1 ΔMTS* (*13-187*)::*gfp*, *cb-unc-119*(+)]; *clk-1*(*qm30*), MQ1993 ukSi2 [*pclk-1*::*clk-1 ΔMTS* (*13-187*)::*gfp*, *cb-unc-119*(+)]; *clk-1*(*e2519*), MQ1968 ukSi2 [*pclk-1*::*clk-1 ΔMTS* (*13-187*)::*gfp*, *cb-unc-119*(+)]; *clk-1*(*qm30*); *daf-2*(*e1370*), MQ1992 *clk-1*(*qm30*); zcls13[*hsp-6*::*GFP*], MQ1990 ukSi1 [*pclk-1*::*clk-1*::*gfp*, *cb-unc-119*(+)]; *clk-1*(*qm30*); zcls13[*hsp-6*::*GFP*], and MQ1991 ukSi2 [*pclk-1*::*clk-1 ΔMTS* (*13-187*)::*gfp*, *cb-unc-119*(+)]; *clk-1*(*qm30*); zcls13[*hsp-6*::*GFP*]. To generate transgenic *clk-1* animals carrying MosI-mediated single copy insertion (MosSCI) of *CLK-1*::*GFP* or *CLK-1*(*ΔMTS*)::*GFP*, *clk-1* mutants were crossed with strains harboring ukSi1 [*pclk-1*::*clk-1*::*gfp*, *cb-unc-119*(+)] or ukSi2 [*pclk-1*::*clk-1 ΔMTS* (*13-187*)::*gfp*, *cb-unc-119*(+)] on chromosome II^18^. The insertions of the transgene were sequenced and the 11 amino acids MTS deletion was confirmed. The reporter strain SJ4100 zcls13 [*hsp-6*::*GFP*] for mitochondrial stress was obtained from the Ceanorhabditis Genetics Center (CGC).

For testing the effects of 2,4-dihydroxybenzoic acid (Sigma-Aldrich W379808) and 3,4-dihydroxybenzoic acid (Sigma-Aldrich 37580) compounds were dissolved in water and the pH was adjusted to 7.4 with NaOH. The appropriate amounts of dihydroxybenzoic acid (DHB) were added to NGM before pouring the plates and the final concentrations are described in the results and Figures. Young adult animals were transferred to the test plates and the effects of compounds were scored after raising the worms on the test plates for one generation.

### Development and behavioural assays

Worms were fed with either the standard *E*. *coli* OP50, which produces UQ_8_, or with GD1 (*ubiG*::Kan)^45^, which is a UQ_8_-deficient *E*. *coli* strain. GD1 was cultured in 2YT medium supplemented with 0.5% glucose and 50ug/ml kanamycin and then was seeded on NGM plates. On day 1, a cleaning procedure was used to transfer worms from OP50 plates to GD1 plates. Firstly, adult worms were transferred to GD1 plates and were left to crawl around for 2 hrs to get rid of any trace amount of OP50. Then the worms were transferred again from these plates to new GD1 plates. Synchronized L1 worms from limited egg-laying were placed onto new plates and were monitored every 24 hr until all of the worms reached adulthood. Animals were scored as adults after they had undergone the final molt and a vulva could be observed. Defecation cycle rates and pharyngeal pumping rate were measured as previously described^11, 46^.

### Lifespan analysis

On day 0, adult worms were transferred to control NGM plates or plates containing DHB for 3 hrs limited egg-laying. Once hatched worms reached L4 larval stage, animals were transferred onto plates containing 50 µM 5-fluorodeoxyuridine (FUdR) which were prepared by adding FUdR from 0.1 M stock solution into NGM media just prior to pouring plates. OP50 grown on regular NGM plates was transferred onto NGM-FUdR plates containing DHB using an inoculating loop instead of seeding directly onto the plates. Control NGM-FUdR plates containing no DHB were treated in the same fashion. Worms were further transferred onto freshly prepared FUdR plates with or without DHB once a week and were monitored until dead. Worms dying from either internal hatching or gut extrusion were not included in the study and were replaced from a backup pool. Differences between survival curves were compared using the log-rank test.

### Quantitative real-time RT-PCR

Synchronized day 1 young adult worms from limited egg-laying were used for total RNA extraction using RNeasy Kit (Qiagen). 1 µg of RNA was converted to cDNA using the Quantitect Reverse Transcription Kit (Qiagen). The resulting cDNA was used for quantitative real-time PCR using the Quantitect SYBR Green PCR Kit (Qiagen) and a Biorad iCycler CFX96 RT-PCR machine. The qRT-PCR amplification cycling conditions were as follows: initial denaturation at 95 °C for 10 min followed by 45 cycles of 94 °C/10 s, 55 °C/10 s and 72 °C/10 s. Results represent the average of at least three independent biological samples and each repeat was run in triplicate. Comparative C_T_ (ΔΔC_T_) method was used to calculate the changes in gene expression. For a valid ΔΔC_T_ calculation, the efficiency of amplification for target and reference genes must be approximately equal. To determine this, amplifications were performed on the same serial diluted samples and efficiencies were determined for each set of primers. PCR efficiencies were close to 100% for all of the primer sets which allowed the use of the ∆∆C_T_ method for calculation of relative gene expression levels. The sequences of primers used are described in Supplementary Table S2.

### Measurement of fluorescent reporter activity

Day 1 young adult worms were synchronized from a limited laying and were used for measuring whole worm florescence. Worms were mounted on 3% agarose pads. A Few drops of 2 mM levamisole were used to paralyze the worms. Florescent images were captured on an Olympus BX63 microscope at 10× magnification using an EXi Blue^TM^ camera. CellSens Dimension version 1.6 software was used for image acquisition and for the measurement of mean gray intensity.

### Quinone extraction and high-performance liquid chromatography

Quinone extraction and quantitation by high-performance liquid chromatography (HPLC) were carried out as previously described^15^. Briefly, worms were lysed in 0.1% SDS for several minutes using a motorized tissue homogenizer and the protein concentration was measured by the BCA assay (Thermo Fisher Scientific). Worm lysates were mixed with equal volume of ethanol and the mixture was vortexed for 30 sec. Then, hexane was added and vortexing continued for 2 more minutes before centrifugation at 2,200 rpm for 5 min to separate layers. The upper organic layer was then transferred to a new tube and hexane was evaporated by drying in a SpeedVac concentrator. The left residual was finally re-dissolved in ethanol before injection into HPLC (a Beckman Coulter System Gold HPLC system with 32 Karat software). The following conditions were employed for HPLC: a reverse phase C18 column (25.0 × 0.46 cm, 5 μm, Highchrom) eluted with mobile phase comprising methanol and ethanol (7:3, v/v) at a flow rate of 1.8 mL/min. Chromatograms were monitored with UV detection at wavelength of 275 nm.

### Subcellular protein fractionation and immunoblot analysis

Cells were fractionated by using a commercial subcellular fractionation kit for cultured cells (Thermo Fisher Scientific) to obtain cytoplasmic, membrane, soluble and insoluble nuclear proteins. Subcellular protein fractionation from mouse tissues was carried out by using the Thermo Scientific^TM^ subcellular protein fractionation kit for tissues according to the manufacturer’s instructions. The proteins in the nuclear and membrane fractions were separated by SDS-PAGE and electrotransferred onto nitrocellulose membranes. The following primary antibodies were used to incubate overnight at 4 °C: anti-HDAC1 (Cell Signaling Technology, 1:1000 dilution), anti-Lamin A/C (Cell Signaling Technology, 1:2000 dilution), anti-SOD2 (Abcam, 1:2000 dilution), anti-Histone H3 (Cell Signaling Technology, 1:1000), and anti-MCLK1 (produced in our laboratory, 1:1000 dilution). After incubation with appropriate HRP-conjugated secondary antibodies, bands were visualized with a chemiluminescence (ECL) reagent (GE Healthcare) followed by exposure to X-ray film.

### Antibody Staining

For fixation, mixed stage worms were washed off plates and washed 3x with M9 buffer. Worms were re-suspended in water and spread evenly across homemade poly-lysine coated slides using a glass pipette. A commercial poly-lysine slide was placed directly on top of the slide containing the worms such that only the unfrosted sections of the slides overlapped. The slide sandwiches were then placed on a flat slab of dry ice for 15 minutes. The slide sandwiches were swiftly twisted apart and the top slide was discarded. The slides were placed back to back in a coplin jar containing ice-cold 100% methanol for 4 minutes. The slides were removed and placed into a coplin jar containing ice-cold 100% acetone for another 4 minutes. The slides were then transferred to a new coplin jar and rinsed indirectly with PBS.

For blocking and staining, the slides were placed in a coplin jar containing block (5% BSA in Antibody Buffer). After 1 hour, the block was poured out and replaced with Primary antibody solution and incubated overnight at 4 °C. After incubation, the Primary antibody solution was poured out of the jar and replaced with PBS. The PBS was exchanged with antibody buffer and placed on a gentle rocker for 30 minutes at room temperature. The antibody buffer was exchanged and incubated for 30 minutes two more times. The antibody buffer was then exchanged for Secondary antibody solution and incubated for 4 hours at room temperature. After incubation, the antibody solution was poured out and replaced with PBS. Immediately, the PBS was replaced with antibody buffer and set at room temperature for 30 minutes. The antibody buffer was exchanged and set for another 30 minutes at room temperature. After incubation, the solution was replaced with PBS. The primary antibodies used were chicken α-GFP (Abcam x) (1:400) and Mouse α-HSP-60 (1:400). The secondary antibodies used were Goat α-Chicken Alexa Fluor488 (Abcam) (1 in 1000) and Goat α-Mouse Alexa Fluor 594(Abcam) (1 in 1000).

For microscopy, the slides were removed from the PBS and the back of the slides were dried using kimwipes. 20 µL of mounting medium (anti-fade and DAPI) was added to each slide and mixed with the residual PBS on the front of the slide. Slides were sealed using 24 × 60 mm coverslips and 2 layers of clear nail polish. Slides were observed immediately on an Olympus BX63 upright microscope equipped with an EXi Blue^TM^ camera and FITC, TxRED and DAPI filters. Images were acquired under 100-1000× magnification using cellSens Dimension (Olympus) software. For quantification, fluorescent micrographs of whole worm sections (n = 20 for each stage and condition) were analyzed using cellSens Dimension and co-localization between different channels was computed to produce Pearson’s Co-localization Coefficient (PCC) values.

### Statistical analyses

Prism software from GraphPad (version 5.0) was used to construct all graphs and perform statistical analyses. Between-group differences, except for survival studies, were analyzed with a Student’s t test, and significant differences are indicated. *P < 0.05; **P < 0.01; ***P < 0.001. Survival was plotted using Kaplan Meier curves and p-values were calculated using the log-rank test.

## Electronic supplementary material

Supplementary Information
