# Supplementary material for: A single biochemical activity underlies the pleiotropy of the aging-related protein CLK-1
Source: Sci Rep. 2017 Apr 12;7:859. doi: 10.1038/s41598-017-00754-z (PMC5429816; doi:10.1038/s41598-017-00754-z)
Supplement: Supplementary file 1 — Supplementary Information [file 41598_2017_754_MOESM1_ESM.pdf]

A single biochemical activity underlies the pleiotropy of the aging-related protein CLK-1

Ju-Ling Liu, Callista Yee, Ying Wang, and Siegfried Hekimi

Figure S1

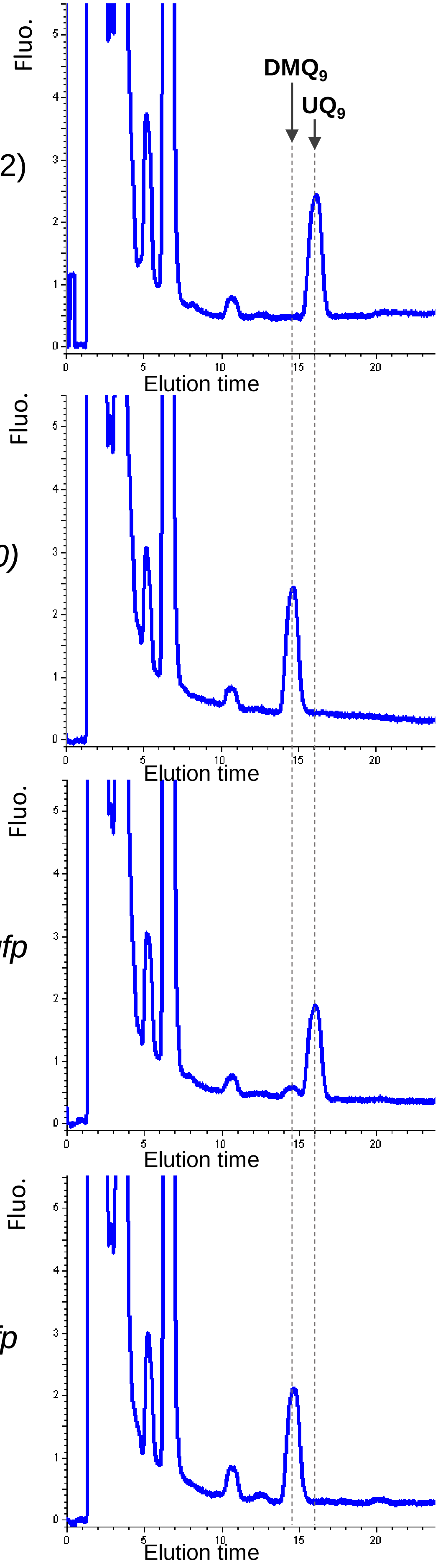

Legend

clk-1(qm30) mutants do not synthesize UQ_9 but instead accumulate DMQ_9, a biosynthetic precursor of UQ_9. The expression of CLK-1::GFP, but not CLK-1(ΔMTS)::GFP rescues the production of UQ_9 in *clk-1(qm30)* mutants.

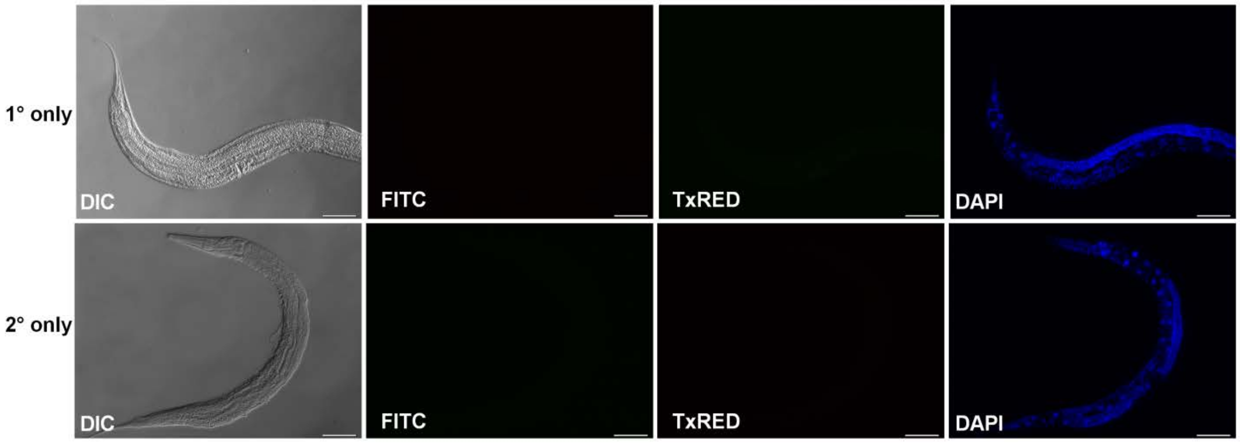

Figure S2 Control staining lacking primary or secondary antibodies. Worms of all stages expressing CLK-1::GFP were freeze-cracked, fixed and stained in solutions lacking either primary or secondary antibody. Slides were mounted with DAPI and observed at 100x. Scale bar in all images is 50 µm.

Figure S3

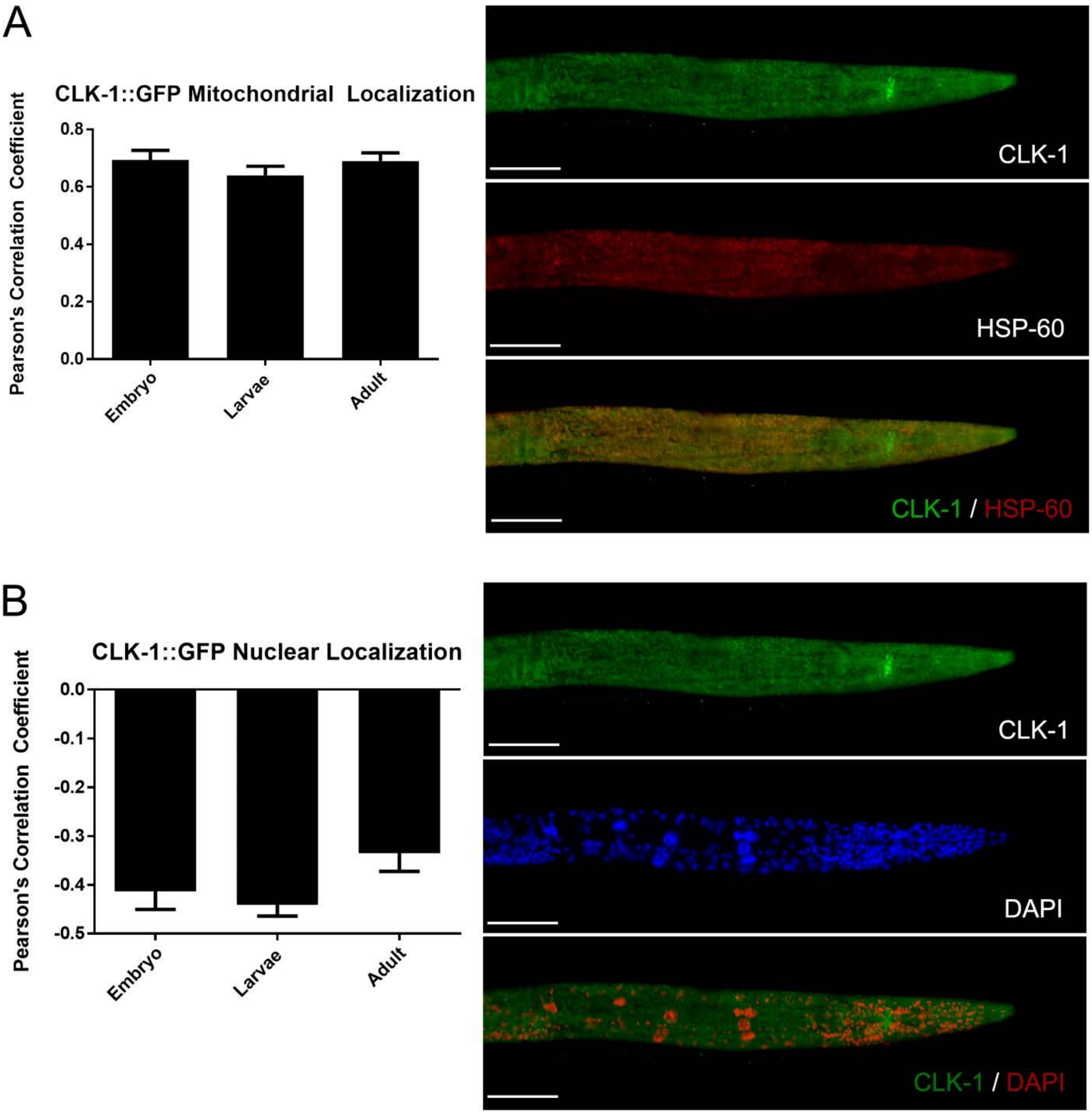

Legend Worms of all stages were freeze-cracked, fixed and stained using antibodies against GFP and HSP-60. A) Left: Quantification of Pearson's Correlation Coefficient between CLK-1::GFP and HSP-60. Values shown are Mean + SEM. Right: Double staining of CLK-1 (FITC filter) and HSP-60 (TxRED filter). B) Left: Quantification of Pearson's Correlation Coefficient between CLK-1::GFP and DAPI. Values shown are Mean + SEM. Right: Double staining of CLK-1 (FITC filter) and DAPI (DAPI filter). Scale bar in all images is 50 µm.

Figure S4

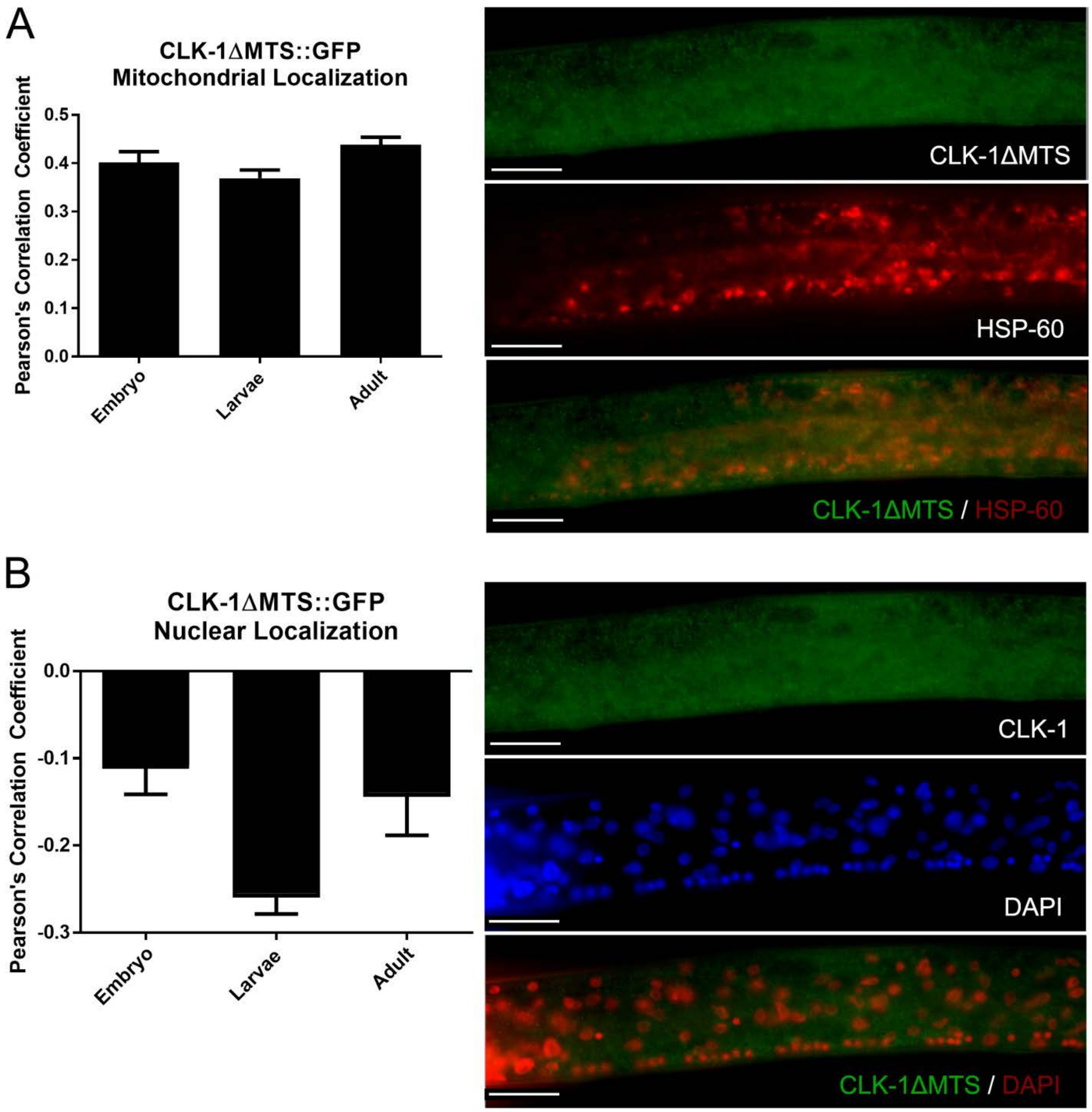

Legend **C**LK-1ΔMTS::GFP Expression is Diffuse but Predominantly Mitochondrial. Worms of all stages were freeze-cracked, fixed and stained using antibodies against GFP and HSP-60. Worms of all stages showed diffuse GFP signal. A) Left: Quantification of Pearson's Correlation Coefficient between CLK-1ΔMTS::GFP and HSP-60. Values shown are Mean + SEM. Right: Double staining of CLK-1ΔMTS (FITC filter) and HSP-60 (TxRED filter). B) Left: Quantification of Pearson's Correlation Coefficient between CLK-1ΔMTS::GFP and DAPI. Values shown are Mean + SEM. Right: Double staining of CLK-1ΔMTS (FITC filter) and DAPI (DAPI filter). Worms shown in this figure were imaged at 400x. Scale bar in all images is 20 μm.

Table S1 Effects of DHB treatment on the growth of wild-type (N2) and *clk-1* mutants fed with UQ-deficient *E. coli*. Synchronized L1 worms were plated on GD1-seeded NGM plates containing different concentrations of DHB (day 0) and left to grow at 20 ºC for 4 days. They were monitored daily for growth and development.

Group	Stages of development			
	**Day 1**	**Day 2**	**Day 3**	**Day 4**
N2	L3	YA	A + progeny (eggs, L1)	A + progeny (larvae, YA)
N2 + 10 mM DHB	L3	YA	A + progeny (eggs, L1)	A + progeny (larvae, YA)
clk-1(qm30)	L2	L2	L2	L2
clk-1(qm30) + 0.01 mM DHB	L2, L3	L2, L3	YA	A
clk-1(qm30) + 0.05 mM DHB	L2, L3	L3, L4	YA, a few A	A + progeny (eggs)
clk-1(qm30) + 0.15 mM DHB	L2, L3	L4, YA	A	A +progeny (eggs+L1)
clk-1(qm30) + 0.5 mM DHB	L3	L4, YA	A + progeny (eggs)	A + progeny (eggs, larvae)
clk-1(qm30) +1 mM DHB	L3	YA	A + progeny (eggs)	A + progeny (larvae, YA)
clk-1(qm30) + 5 mM DHB	L3	YA	A + progeny (eggs, L1)	A + progeny (larvae, YA)

L1, L2, L3 and L4, larval stages; YA, young adult; A, adults with eggs.

Table S2 Individual aging, defecation and pumping rate experiments and statistics

Genotype	Treatment	Mean lifespan (days ±S.D.)	Maximum lifespan (days)	p-values vs. control (log-rank test)	Mean lifespan changes in days
Wild Type	Control	18.1 ± 1.8(n=75)	24		
	0.15mM 2,4-DHB	18.1 ± 2.0(n=75)	25		
	1mM 2,4-DHB	17.8 ± 2.0(n=75)	23		
clk-1(qm30)	Control	25.0 ± 3.1(n=75)	33		
	0.15mM 2,4-DHB	18.3 ± 2.6(n=75)	33	P<0.0001	-6.7
	1mM 2,4-DHB	17.8 ± 2.0(n=75)	24	P<0.0001	-7.2
clk-1(e2519)	Control	21.6 ± 1.9(n=50)	27		
	1mM 2,4-DHB	18.2 ± 2.1(n=50)	22	P<0.0001	-3.4
Wild Type	Control	17.2 ± 2.3(n=50)	25		
	1mM 3,4-DHB	16.4 ± 1.9(n=50)	21		
clk-1(qm30)	Control	22.5 ± 4.1(n=100)	35		
	1mM 3,4-DHB	21.7 ± 3.3(n=100)	30		
clk-1(qm30)	Control	24.6 ± 2.4(n=100)	31		
clk-1(qm30); CLK-1(ΔMTS)::GFP		24.4 ± 3.0(n=100)	33		
clk-1(qm30); daf-2(e1370)	Control	56.3 ± 15.4(n=50)	78		
clk-1(qm30); daf-2(e1370); CLK-1(ΔMTS)::GFP		56.9 ± 14.3(n=50)	80		
clk-1(e2519)	Control	21.3 ± 2.3(n=50)	26		
clk-1(e2519); CLK-1(ΔMTS)::GFP		21.4 ± 1.7(n=50)	24		
Wild Type	Control	17.2 ± 2.3(n=50)	24		
	1mM 2,4-DHB	17.1 ± 2.0(n=50)	24		
clk-1(qm30); CLK-1(ΔMTS)::GFP	Control	23.7 ± 2.8(n=50)	30		
	1mM 2,4-DHB	16.4 ± 2.4(n=50)	21	P<0.0001	-7.3

Table S2, continued

Genotype	Treatment	Mean defecation cycle length (sec) Mean ± SEM	Maximum defecation cycle length (sec)	p-values vs. control (t-test)	Mean defecation cycle length change (sec)
Wild type	Control	55.7 ± 0.6(n=20)	60.0		
	1mM 2,4-DHB	55.8 ± 0.6(n=20)	60.0		
	10mM 2,4-DHB	55.1 ± 0.5(n=22)	59.8		
clk-1(qm30)	Control	96.9 ± 2.1(n=20)	109.0		
	1mM 2,4-DHB	57.5 ± 0.5(n=22)	60.4	P<0.0001	-39.4
	10mM 2,4-DHB	55.7 ± 0.7(n=21)	59.6	P<0.0001	-41.2
Wild type	Control	53.0 ± 0.4(n=20)	56.0		
clk-1(qm30)		89.6 ± 3.2(n=22)	122.0	P<0.0001	+36.6
clk-1(qm30); CLK-1::GFP		53.3 ± 0.5(n=22)	57.0		
clk-1(qm30); CLK-1(ΔMTS)::GFP		94.5 ± 3.4(n=21)	125.3	P<0.0001	+41.5

Genotype	Treatment	Mean number of pumps per minute Mean ± SEM	Maximum number of pumps per minute	p-values vs. control (t-test)	Mean number of pumps change
Wild type	Control	292.7 ± 5.2(n=22)	336		
	1mM 2,4-DHB	297.2 ± 4.9(n=20)	342		
	10mM 2,4-DHB	291.5 ± 4.3(n=22)	336		
clk-1(qm30)	Control	180.6 ± 3.7(n=22)	212		
	1mM 2,4-DHB	297.6 ± 5.5(n=21)	336	P<0.0001	+117.0
	10mM 2,4-DHB	296.5 ± 6.2(n=21)	356	P<0.0001	+115.9
Wild type	Control	297.0 ± 5.2(n=22)	358		
clk-1(qm30)		182.7 ± 3.5(n=22)	218	P<0.0001	-114.3
clk-1(qm30); CLK-1::GFP		302.5 ± 4.7(n=22)	338		
clk-1(qm30); CLK-1(ΔMTS)::GFP		183.8 ± 3.6(n=22)	210	P<0.0001	-113.2

Table S3 Real-time PCR primers

**Gene**	**Forward primer sequences**	**Reverse primer sequences**	**Size(bp)**
pmp-3 (Control)*	GTTCCCGTGTTCATCACTCAT	ACACCGTCGAGAAGCTGTAGA	116
hsp-60	GTCTTGAGCCATCGTCGATTATTG	CTGTGCGAACCACCTTAGTTGG	136
cdc-42 (Control)*	CTGCTGGACAGGAAGATTACG	CTCGGACATTCTCGAATGAAG	112
hsp-6	GGAACAACAGATCGTTATCCAATC	GTGTCGTGGATGATTCCTTCAG	153
spg-7	CAATTCCCAGGAGGATGGC	CTTCAAGTCTCTCGACAAGTCCAG	151

*The reference gene for each target gene was assessed by a validation experiment to test whether they share equal amplification efficiency.
